# 2-[2-(2-Bromo­phen­yl)-2-oxoeth­yl]-1λ^6^,2-benzothia­zole-1,1,3-trione

**DOI:** 10.1107/S1600536812022428

**Published:** 2012-05-26

**Authors:** Nazia Sattar, Hamid Latif Siddiqui, Waseeq Ahmad Siddiqui, Muhammad Akram, Masood Parvez

**Affiliations:** a Institute of Chemistry, University of the Punjab, Lahore 54590, Pakistan; b Department of Chemistry, University of Sargodha, Sargodha 40100, Pakistan; c Department of Chemistry, Universiti Teknologi Malaysia, 81310 UTM Skudai Johor, Darul Ta’zim, Malaysia; d Department of Chemistry, The University of Calgary, 2500 University Drive NW, Calgary, Alberta, Canada T2N 1N4

## Abstract

The asymmetric unit of the title compound, C_15_H_10_BrNO_4_S, contains two different conformers in which the benzisothia­zole rings are essentially planar, with r.m.s. deviations of 0.012 and 0.017 Å. The mean planes of the benzene rings form dihedral angles 70.49 (13) and 72.79 (11)° with the benzisothia­zole rings. The orientation of the Br atoms in the two conformers exhibit the most pronounced difference, with opposing orientations in the two mol­ecules. The crystal structure is stabilized by π–π inter­actions between the benzene rings of the benzisothia­zole moieties of one mol­ecule and bromo­benzene rings of the other mol­ecule, with distances between the ring centroids of 3.599 (3) and 3.620 (3) Å, respectively. The crystal packing is further consolidated by pairs of weak inter­molecular C—H⋯O hydrogen bonds, which form inversion dimers.

## Related literature

For non-steroidal anti-inflammatory drugs (NSAIDs) and related compounds, see: Lombardino *et al.* (1971 ▶); Soler (1985 ▶); Carty *et al.* (1993 ▶); Turck *et al.* (1995 ▶); Blackham & Owen (1975 ▶); Singh *et al.* (2007 ▶); Vaccarino *et al.* (2007 ▶); Kapui *et al.* (2003 ▶). For related structures, see: Maliha *et al.* (2007 ▶); Siddiqui *et al.* (2007 ▶).
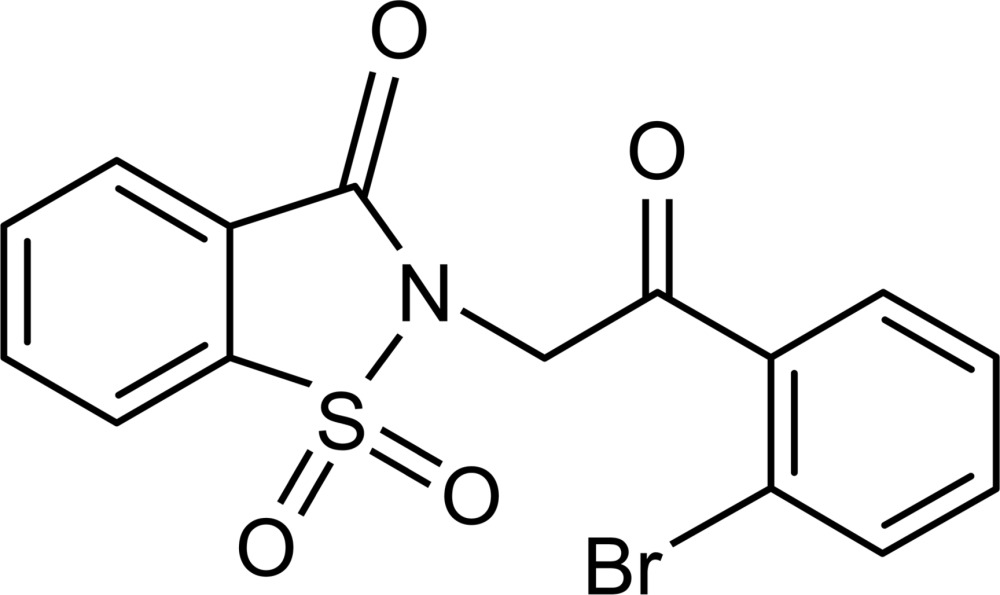


## Experimental

### 

#### Crystal data

C_15_H_10_BrNO_4_S
*M* *_r_* = 380.21
Triclinic, 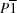

*a* = 7.574 (2) Å
*b* = 13.903 (4) Å
*c* = 14.814 (4) Å
α = 110.574 (15)°
β = 96.936 (13)°
γ = 93.640 (14)°
*V* = 1440.3 (7) Å^3^
*Z* = 4
Mo *K*α radiation
μ = 3.02 mm^−1^
*T* = 123 K
0.18 × 0.18 × 0.16 mm

#### Data collection

Nonius KappaCCD diffractometer
Absorption correction: multi-scan (*SORTAV*; Blessing, 1997 ▶) *T*_min_ = 0.613, *T*_max_ = 0.644
12284 measured reflections
6541 independent reflections
5268 reflections with *I* > 2σ(*I*)
*R*_int_ = 0.042

#### Refinement

*R*[*F* ^2^ > 2σ(*F* ^2^)] = 0.050
*wR*(*F* ^2^) = 0.105
*S* = 1.12
6541 reflections
397 parameters
H-atom parameters constrained
Δρ_max_ = 0.81 e Å^−3^
Δρ_min_ = −1.04 e Å^−3^

### 

Data collection: *COLLECT* (Hooft, 1998 ▶); cell refinement: *DENZO* (Otwinowski & Minor, 1997 ▶); data reduction: *SCALEPACK* (Otwinowski & Minor, 1997 ▶); program(s) used to solve structure: *SHELXS97* (Sheldrick, 2008 ▶); program(s) used to refine structure: *SHELXL97* (Sheldrick, 2008 ▶); molecular graphics: *ORTEP-3 for Windows* (Farrugia, 1997 ▶); software used to prepare material for publication: *SHELXL97*.

## Supplementary Material

Crystal structure: contains datablock(s) global, I. DOI: 10.1107/S1600536812022428/pk2412sup1.cif

Structure factors: contains datablock(s) I. DOI: 10.1107/S1600536812022428/pk2412Isup2.hkl

Supplementary material file. DOI: 10.1107/S1600536812022428/pk2412Isup3.cml

Additional supplementary materials:  crystallographic information; 3D view; checkCIF report

## Figures and Tables

**Table 1:** Hydrogen-bond geometry (Å, °)

*D*—H⋯*A*	*D*—H	H⋯*A*	*D*⋯*A*	*D*—H⋯*A*
C14—H14⋯O1^i^	0.95	2.40	3.305 (5)	159
C17—H17⋯O5^ii^	0.95	2.43	3.225 (5)	141
C27—H27⋯O7^iii^	0.95	2.29	3.164 (5)	153

Symmetry codes: (i) 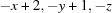; (ii) 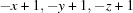; (iii) 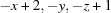.

**Fig. 1.**
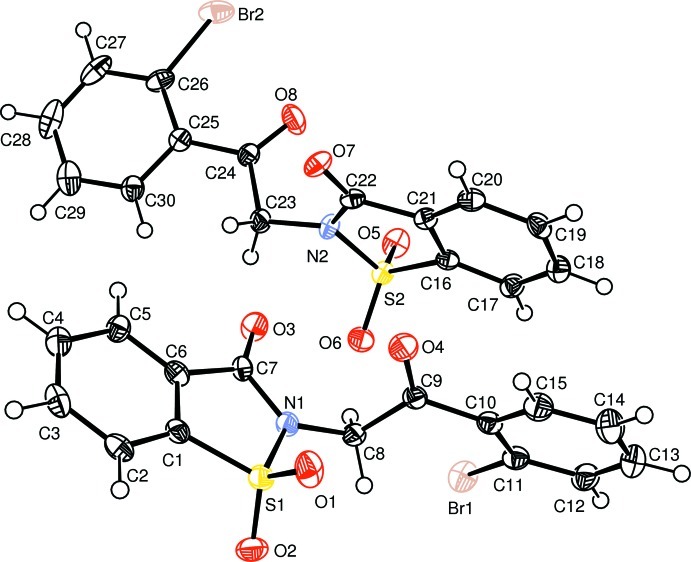
The asymmetric unit of the title compound. Displacement ellipsoids are drawn at the 50% probability level. H atoms are presented as small spheres of arbitrary radius.

**Fig. 2.**
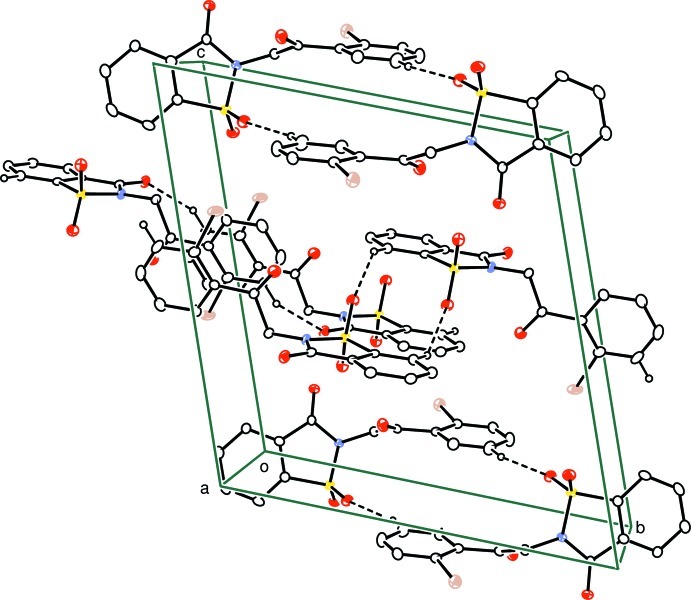
A view of the C—H···O hydrogen bonds (dotted lines) in the crystal structure of the title compound. H atoms not participating in hydrogen-bonding were omitted to enhance clarity.

## supplementary crystallographic information

### Comment

Oxicam, a class of non-steroidal anti-inflammatory drugs (NSAIDs) consists of 1,2-benzothiazine 1,1-dioxide derivatives which are found to be potent anti-inflammatory and analgesic agents, *e.g.*, Piroxicam (Lombardino *et al.*, 1971), Droxicam (Soler, 1985), Ampiroxicam (Carty *et al.*, 1993), Meloxicam (Turck *et al.*, 1995) and Sudoxicam (Blackham & Owen, 1975) are the recent members of this class currently in use in the international market. Various derivatives are known to be cyclooxygenase-2 (COX-2) inhibitors (Singh *et al.*, 2007), analgesic (Vaccarino *et al.*, 2007) and human leucocyte elastase (HLE) inhibitors (Kapui *et al.*, 2003). Earlier, we have reported the synthesis and crystal structures of some of the 1,2-benzothiazine derivatives (Maliha *et al.*, 2007; Siddiqui *et al.*, 2007). Herein, we report the synthesis and crystal structure of the title compound that has served as a precursor for the 1,2-benzothiazine derivative.

The asymmetric unit of the title compound contains two conformers (Fig. 1). In both molecules, the benzisothiazol rings S1/N1/C1–C7 and S2/N2/C16–C22 are essentially planar with rms deviations of fitted atoms being 0.017 Å and 0.012 Å, respectively, while the mean-planes of the benzene rings C10–C15 and C25–C30 form dihedral angles 70.49 (13)° and 72.79 (11)°, respectively, with the mean-planes of the benzisothiazol rings. The orientation of the Br atoms in the two conformers exhibit the most pronounced difference, with opposing orientations in the two molecules. The crystal structure is stabilized by π-π interactions between benzene rings (C1–C6) of benzisothiazol moieties in one molecule and bromobenzene rings (C25–C30) in the other molecule with distances between the ring centroids being 3.599 (3) Å and 3.620 (3) Å, respectively. The crystal packing is further consolidated by weak intermolecular C—H···O hydrogen bonds. The molecule containing S1 forms centrosymmetric dimers *via* C14—H14···O1 hydrogen bonding interactions. The other molecule also forms centrosymmetric dimers *via* C17—H17···O5 hydrogen bonds; the dimers are further extended along the *b*-axis *via* C27—H27···O7 hydrogen bonds (Fig. 2 and Tab. 1).

The bond distances and angles in the title compound (Fig. 1) agree very well with the corresponding bond distances and angles reported in closely related compounds (Maliha *et al.*, 2007; Siddiqui *et al.*, 2007).

### Experimental

A mixture of 2-bromo-1-(2-bromophenyl)ethanone (2.0 g, 7.2 mmol) and sodium saccharin (1.76 g, 8.6 mmol) in dimethylformamide (15 ml) was stirred at 383 K for 3 h under anhydrous conditions. On completion of the reaction (as indicated by TLC), the contents of the flask were poured into crushed ice. The precipitates formed were filtered, washed with water, ice-cold ethanol and dried to give the reddish brown title product (1.94 g, 71%). Crystals were grown by slow evaporation of a solution in EtOAc and CHCl_3_ (1:1) at room temperature; m.p. 395–397 K.

### Refinement

All H atoms were positioned geometrically and refined using a riding model, with C—H = 0.95 and 0.99 Å, for aryl and methylene H-atoms, respectively. The *U*_iso_(H) were allowed at 1.2*U*_eq_(C).

### Crystal data

**Table:**

C_15_H_10_BrNO_4_S	*Z* = 4
*M**_r_* = 380.21	*F*(000) = 760
Triclinic, *P*1	*D*_x_ = 1.753 Mg m^−^^3^
Hall symbol: -P 1	Mo *K*α radiation, λ = 0.71073 Å
*a* = 7.574 (2) Å	Cell parameters from 6425 reflections
*b* = 13.903 (4) Å	θ = 1.0–27.5°
*c* = 14.814 (4) Å	µ = 3.02 mm^−^^1^
α = 110.574 (15)°	*T* = 123 K
β = 96.936 (13)°	Prism, colorless
γ = 93.640 (14)°	0.18 × 0.18 × 0.16 mm
*V* = 1440.3 (7) Å^3^	

### Data collection

**Table:**

Nonius KappaCCD diffractometer	6541 independent reflections
Radiation source: fine-focus sealed tube	5268 reflections with *I* > 2σ(*I*)
Graphite monochromator	*R*_int_ = 0.042
ω and φ scans	θ_max_ = 27.5°, θ_min_ = 1.5°
Absorption correction: multi-scan (*SORTAV*; Blessing, 1997)	*h* = −9→9
*T*_min_ = 0.613, *T*_max_ = 0.644	*k* = −18→18
12284 measured reflections	*l* = −19→19

### Refinement

**Table:**

Refinement on *F*^2^	Primary atom site location: structure-invariant direct methods
Least-squares matrix: full	Secondary atom site location: difference Fourier map
*R*[*F*^2^ > 2σ(*F*^2^)] = 0.050	Hydrogen site location: difference Fourier map
*wR*(*F*^2^) = 0.105	H-atom parameters constrained
*S* = 1.12	*w* = 1/[σ^2^(*F*_o_^2^) + 5.440*P*] where *P* = (*F*_o_^2^ + 2*F*_c_^2^)/3
6541 reflections	(Δ/σ)_max_ < 0.001
397 parameters	Δρ_max_ = 0.81 e Å^−^^3^
0 restraints	Δρ_min_ = −1.04 e Å^−^^3^

### Special details

**Table:**

Geometry. All e.s.d.'s (except the e.s.d. in the dihedral angle between two l.s. planes) are estimated using the full covariance matrix. The cell e.s.d.'s are taken into account individually in the estimation of e.s.d.'s in distances, angles and torsion angles; correlations between e.s.d.'s in cell parameters are only used when they are defined by crystal symmetry. An approximate (isotropic) treatment of cell e.s.d.'s is used for estimating e.s.d.'s involving l.s. planes.
Refinement. Refinement of *F*^2^ against ALL reflections. The weighted *R*-factor *wR* and goodness of fit *S* are based on *F*^2^, conventional *R*-factors *R* are based on *F*, with *F* set to zero for negative *F*^2^. The threshold expression of *F*^2^ > σ(*F*^2^) is used only for calculating *R*-factors(gt) *etc*. and is not relevant to the choice of reflections for refinement. *R*-factors based on *F*^2^ are statistically about twice as large as those based on *F*, and *R*- factors based on ALL data will be even larger.

### Fractional atomic coordinates and isotropic or equivalent isotropic displacement parameters (Å^2^)

**Table:**

	*x*	*y*	*z*	*U*_iso_*/*U*_eq_	
Br1	0.36505 (6)	0.45967 (4)	0.15933 (3)	0.03241 (12)	
Br2	0.91717 (7)	0.09057 (4)	0.66368 (3)	0.03844 (13)	
S1	0.67803 (14)	0.13040 (8)	−0.09125 (7)	0.0234 (2)	
S2	0.60738 (13)	0.34637 (7)	0.39090 (7)	0.02000 (19)	
O1	0.8217 (4)	0.1917 (2)	−0.1062 (2)	0.0349 (7)	
O2	0.5086 (4)	0.1188 (2)	−0.1509 (2)	0.0321 (7)	
O3	0.6920 (4)	0.1327 (2)	0.1629 (2)	0.0283 (6)	
O4	0.9038 (4)	0.3378 (2)	0.1237 (2)	0.0310 (7)	
O5	0.5563 (4)	0.3780 (2)	0.4859 (2)	0.0283 (6)	
O6	0.4665 (4)	0.3119 (2)	0.3092 (2)	0.0283 (6)	
O7	1.0338 (4)	0.2269 (2)	0.3508 (2)	0.0260 (6)	
O8	0.8207 (5)	0.2096 (2)	0.5384 (2)	0.0389 (8)	
N1	0.6521 (5)	0.1750 (2)	0.0257 (2)	0.0215 (7)	
N2	0.7478 (4)	0.2569 (2)	0.3791 (2)	0.0207 (7)	
C1	0.7402 (5)	0.0120 (3)	−0.0868 (3)	0.0209 (8)	
C2	0.7806 (5)	−0.0711 (3)	−0.1628 (3)	0.0250 (8)	
H2	0.7750	−0.0703	−0.2270	0.030*	
C3	0.8297 (5)	−0.1558 (3)	−0.1406 (3)	0.0277 (9)	
H3	0.8576	−0.2148	−0.1908	0.033*	
C4	0.8388 (5)	−0.1557 (3)	−0.0463 (3)	0.0261 (9)	
H4	0.8734	−0.2146	−0.0332	0.031*	
C5	0.7983 (5)	−0.0711 (3)	0.0290 (3)	0.0230 (8)	
H5	0.8054	−0.0711	0.0934	0.028*	
C6	0.7473 (5)	0.0133 (3)	0.0077 (3)	0.0208 (8)	
C7	0.6977 (5)	0.1109 (3)	0.0771 (3)	0.0198 (8)	
C8	0.5961 (5)	0.2769 (3)	0.0709 (3)	0.0213 (8)	
H8A	0.5346	0.2778	0.1264	0.026*	
H8B	0.5100	0.2916	0.0229	0.026*	
C9	0.7555 (5)	0.3606 (3)	0.1071 (3)	0.0205 (8)	
C10	0.7307 (5)	0.4707 (3)	0.1184 (3)	0.0215 (8)	
C11	0.5795 (5)	0.5213 (3)	0.1368 (3)	0.0234 (8)	
C12	0.5781 (6)	0.6246 (3)	0.1462 (3)	0.0305 (9)	
H12	0.4728	0.6577	0.1583	0.037*	
C13	0.7319 (7)	0.6784 (4)	0.1378 (4)	0.0384 (11)	
H13	0.7330	0.7492	0.1453	0.046*	
C14	0.8831 (7)	0.6296 (4)	0.1187 (4)	0.0386 (11)	
H14	0.9878	0.6663	0.1118	0.046*	
C15	0.8825 (6)	0.5280 (3)	0.1095 (3)	0.0290 (9)	
H15	0.9880	0.4953	0.0967	0.035*	
C16	0.7715 (5)	0.4356 (3)	0.3809 (3)	0.0181 (7)	
C17	0.7503 (5)	0.5350 (3)	0.3834 (3)	0.0229 (8)	
H17	0.6394	0.5629	0.3918	0.028*	
C18	0.8992 (5)	0.5917 (3)	0.3730 (3)	0.0249 (8)	
H18	0.8906	0.6597	0.3735	0.030*	
C19	1.0615 (5)	0.5497 (3)	0.3619 (3)	0.0232 (8)	
H19	1.1622	0.5905	0.3564	0.028*	
C20	1.0793 (5)	0.4497 (3)	0.3586 (3)	0.0226 (8)	
H20	1.1896	0.4213	0.3501	0.027*	
C21	0.9302 (5)	0.3926 (3)	0.3681 (3)	0.0181 (7)	
C22	0.9183 (5)	0.2841 (3)	0.3645 (3)	0.0197 (8)	
C23	0.6895 (6)	0.1524 (3)	0.3720 (3)	0.0224 (8)	
H23A	0.7401	0.1020	0.3186	0.027*	
H23B	0.5574	0.1393	0.3561	0.027*	
C24	0.7505 (5)	0.1370 (3)	0.4683 (3)	0.0204 (8)	
C25	0.7189 (5)	0.0296 (3)	0.4674 (3)	0.0196 (8)	
C26	0.7853 (5)	−0.0009 (3)	0.5443 (3)	0.0255 (9)	
C27	0.7537 (6)	−0.1035 (4)	0.5369 (4)	0.0343 (11)	
H27	0.8041	−0.1240	0.5883	0.041*	
C28	0.6490 (7)	−0.1757 (4)	0.4549 (4)	0.0383 (12)	
H28	0.6255	−0.2452	0.4508	0.046*	
C29	0.5792 (6)	−0.1468 (3)	0.3795 (3)	0.0335 (10)	
H29	0.5064	−0.1961	0.3236	0.040*	
C30	0.6150 (5)	−0.0461 (3)	0.3851 (3)	0.0243 (8)	
H30	0.5682	−0.0275	0.3319	0.029*	

### Atomic displacement parameters (Å^2^)

**Table:**

	*U*^11^	*U*^22^	*U*^33^	*U*^12^	*U*^13^	*U*^23^
Br1	0.0246 (2)	0.0352 (2)	0.0418 (3)	0.01061 (18)	0.01351 (19)	0.0153 (2)
Br2	0.0388 (3)	0.0569 (3)	0.0219 (2)	0.0144 (2)	0.00103 (19)	0.0167 (2)
S1	0.0299 (5)	0.0228 (5)	0.0171 (4)	0.0017 (4)	0.0034 (4)	0.0071 (4)
S2	0.0177 (4)	0.0206 (5)	0.0245 (5)	0.0040 (4)	0.0070 (4)	0.0101 (4)
O1	0.048 (2)	0.0283 (16)	0.0300 (16)	−0.0069 (14)	0.0119 (15)	0.0128 (14)
O2	0.0371 (18)	0.0359 (17)	0.0229 (15)	0.0102 (14)	−0.0017 (13)	0.0111 (13)
O3	0.0403 (18)	0.0268 (15)	0.0198 (14)	0.0082 (13)	0.0104 (13)	0.0084 (12)
O4	0.0248 (16)	0.0288 (16)	0.0371 (17)	0.0087 (13)	0.0015 (13)	0.0092 (14)
O5	0.0278 (16)	0.0325 (16)	0.0313 (16)	0.0069 (13)	0.0172 (13)	0.0151 (13)
O6	0.0202 (14)	0.0290 (16)	0.0341 (16)	0.0009 (12)	0.0004 (12)	0.0111 (13)
O7	0.0233 (15)	0.0285 (15)	0.0328 (16)	0.0122 (12)	0.0074 (12)	0.0166 (13)
O8	0.059 (2)	0.0251 (16)	0.0254 (16)	−0.0119 (15)	−0.0055 (15)	0.0068 (13)
N1	0.0300 (18)	0.0185 (16)	0.0148 (15)	0.0028 (14)	0.0034 (14)	0.0044 (13)
N2	0.0239 (17)	0.0175 (16)	0.0249 (17)	0.0046 (13)	0.0076 (14)	0.0110 (14)
C1	0.0184 (19)	0.0199 (19)	0.0218 (19)	−0.0026 (15)	−0.0003 (15)	0.0061 (16)
C2	0.025 (2)	0.028 (2)	0.0169 (18)	−0.0004 (17)	0.0037 (16)	0.0019 (16)
C3	0.023 (2)	0.021 (2)	0.031 (2)	−0.0006 (16)	0.0070 (17)	−0.0009 (17)
C4	0.0187 (19)	0.022 (2)	0.035 (2)	0.0026 (16)	0.0054 (17)	0.0076 (18)
C5	0.023 (2)	0.0214 (19)	0.027 (2)	0.0023 (16)	0.0058 (16)	0.0105 (17)
C6	0.0180 (19)	0.0217 (19)	0.0202 (18)	−0.0023 (15)	0.0034 (15)	0.0052 (16)
C7	0.0206 (19)	0.0157 (18)	0.0222 (19)	−0.0009 (14)	0.0029 (15)	0.0065 (15)
C8	0.0207 (19)	0.0203 (19)	0.0217 (19)	0.0044 (15)	0.0010 (15)	0.0067 (16)
C9	0.024 (2)	0.0204 (19)	0.0158 (17)	0.0051 (15)	0.0028 (15)	0.0039 (15)
C10	0.022 (2)	0.024 (2)	0.0174 (18)	0.0013 (16)	0.0015 (15)	0.0072 (16)
C11	0.023 (2)	0.027 (2)	0.0204 (19)	0.0049 (16)	0.0032 (16)	0.0079 (16)
C12	0.036 (2)	0.026 (2)	0.028 (2)	0.0116 (19)	0.0030 (19)	0.0074 (18)
C13	0.046 (3)	0.023 (2)	0.045 (3)	0.004 (2)	0.001 (2)	0.012 (2)
C14	0.036 (3)	0.034 (3)	0.047 (3)	−0.008 (2)	0.003 (2)	0.019 (2)
C15	0.023 (2)	0.031 (2)	0.033 (2)	0.0036 (18)	0.0031 (18)	0.0122 (19)
C16	0.0201 (19)	0.0215 (19)	0.0129 (16)	0.0022 (15)	0.0024 (14)	0.0064 (15)
C17	0.024 (2)	0.023 (2)	0.0229 (19)	0.0068 (16)	0.0046 (16)	0.0084 (16)
C18	0.027 (2)	0.022 (2)	0.026 (2)	0.0015 (16)	0.0060 (17)	0.0087 (17)
C19	0.022 (2)	0.024 (2)	0.0216 (19)	−0.0064 (16)	0.0027 (16)	0.0085 (16)
C20	0.0170 (19)	0.029 (2)	0.0229 (19)	0.0040 (16)	0.0035 (15)	0.0101 (17)
C21	0.0193 (18)	0.0208 (18)	0.0149 (17)	0.0044 (15)	0.0045 (14)	0.0066 (15)
C22	0.0201 (19)	0.025 (2)	0.0159 (17)	0.0047 (15)	0.0016 (15)	0.0093 (15)
C23	0.029 (2)	0.0188 (19)	0.0212 (19)	−0.0011 (16)	0.0027 (16)	0.0097 (16)
C24	0.0211 (19)	0.0204 (19)	0.0224 (19)	0.0027 (15)	0.0052 (15)	0.0102 (16)
C25	0.0195 (19)	0.0214 (19)	0.0191 (18)	0.0043 (15)	0.0069 (15)	0.0070 (15)
C26	0.022 (2)	0.035 (2)	0.026 (2)	0.0116 (17)	0.0077 (16)	0.0154 (18)
C27	0.038 (3)	0.042 (3)	0.043 (3)	0.022 (2)	0.021 (2)	0.032 (2)
C28	0.047 (3)	0.026 (2)	0.056 (3)	0.015 (2)	0.033 (3)	0.022 (2)
C29	0.037 (3)	0.025 (2)	0.038 (3)	0.0022 (19)	0.018 (2)	0.007 (2)
C30	0.023 (2)	0.022 (2)	0.027 (2)	0.0052 (16)	0.0077 (17)	0.0073 (17)

### Geometric parameters (Å, º)

**Table:**

Br1—C11	1.908 (4)	C10—C15	1.403 (6)
Br2—C26	1.890 (4)	C11—C12	1.394 (6)
S1—O1	1.426 (3)	C12—C13	1.387 (7)
S1—O2	1.433 (3)	C12—H12	0.9500
S1—N1	1.664 (3)	C13—C14	1.377 (7)
S1—C1	1.762 (4)	C13—H13	0.9500
S2—O5	1.429 (3)	C14—C15	1.370 (6)
S2—O6	1.431 (3)	C14—H14	0.9500
S2—N2	1.664 (3)	C15—H15	0.9500
S2—C16	1.757 (4)	C16—C21	1.380 (5)
O3—C7	1.206 (5)	C16—C17	1.389 (5)
O4—C9	1.206 (5)	C17—C18	1.390 (5)
O7—C22	1.207 (4)	C17—H17	0.9500
O8—C24	1.200 (5)	C18—C19	1.397 (6)
N1—C7	1.395 (5)	C18—H18	0.9500
N1—C8	1.453 (5)	C19—C20	1.390 (5)
N2—C22	1.386 (5)	C19—H19	0.9500
N2—C23	1.455 (5)	C20—C21	1.388 (5)
C1—C2	1.382 (5)	C20—H20	0.9500
C1—C6	1.387 (5)	C21—C22	1.487 (5)
C2—C3	1.390 (6)	C23—C24	1.537 (5)
C2—H2	0.9500	C23—H23A	0.9900
C3—C4	1.389 (6)	C23—H23B	0.9900
C3—H3	0.9500	C24—C25	1.493 (5)
C4—C5	1.389 (6)	C25—C26	1.400 (5)
C4—H4	0.9500	C25—C30	1.405 (6)
C5—C6	1.383 (5)	C26—C27	1.394 (6)
C5—H5	0.9500	C27—C28	1.387 (7)
C6—C7	1.489 (5)	C27—H27	0.9500
C8—C9	1.525 (5)	C28—C29	1.373 (7)
C8—H8A	0.9900	C28—H28	0.9500
C8—H8B	0.9900	C29—C30	1.381 (6)
C9—C10	1.507 (5)	C29—H29	0.9500
C10—C11	1.387 (5)	C30—H30	0.9500
			
O1—S1—O2	116.92 (19)	C14—C13—C12	120.2 (4)
O1—S1—N1	109.81 (18)	C14—C13—H13	119.9
O2—S1—N1	109.41 (18)	C12—C13—H13	119.9
O1—S1—C1	112.50 (19)	C15—C14—C13	119.9 (4)
O2—S1—C1	112.67 (18)	C15—C14—H14	120.1
N1—S1—C1	92.85 (17)	C13—C14—H14	120.1
O5—S2—O6	117.20 (18)	C14—C15—C10	122.0 (4)
O5—S2—N2	109.94 (17)	C14—C15—H15	119.0
O6—S2—N2	109.76 (18)	C10—C15—H15	119.0
O5—S2—C16	112.41 (18)	C21—C16—C17	123.2 (3)
O6—S2—C16	112.30 (17)	C21—C16—S2	110.1 (3)
N2—S2—C16	92.44 (17)	C17—C16—S2	126.7 (3)
C7—N1—C8	123.3 (3)	C16—C17—C18	116.7 (4)
C7—N1—S1	115.4 (3)	C16—C17—H17	121.7
C8—N1—S1	121.2 (3)	C18—C17—H17	121.7
C22—N2—C23	121.7 (3)	C17—C18—C19	120.7 (4)
C22—N2—S2	115.7 (3)	C17—C18—H18	119.7
C23—N2—S2	122.2 (3)	C19—C18—H18	119.7
C2—C1—C6	123.1 (4)	C20—C19—C18	121.7 (4)
C2—C1—S1	127.2 (3)	C20—C19—H19	119.2
C6—C1—S1	109.6 (3)	C18—C19—H19	119.2
C1—C2—C3	116.5 (4)	C21—C20—C19	117.7 (4)
C1—C2—H2	121.7	C21—C20—H20	121.1
C3—C2—H2	121.7	C19—C20—H20	121.1
C4—C3—C2	121.3 (4)	C16—C21—C20	120.1 (3)
C4—C3—H3	119.4	C16—C21—C22	113.3 (3)
C2—C3—H3	119.4	C20—C21—C22	126.6 (3)
C3—C4—C5	121.1 (4)	O7—C22—N2	124.1 (4)
C3—C4—H4	119.5	O7—C22—C21	127.4 (4)
C5—C4—H4	119.5	N2—C22—C21	108.4 (3)
C6—C5—C4	118.3 (4)	N2—C23—C24	110.8 (3)
C6—C5—H5	120.9	N2—C23—H23A	109.5
C4—C5—H5	120.9	C24—C23—H23A	109.5
C5—C6—C1	119.7 (4)	N2—C23—H23B	109.5
C5—C6—C7	126.8 (4)	C24—C23—H23B	109.5
C1—C6—C7	113.5 (3)	H23A—C23—H23B	108.1
O3—C7—N1	124.0 (4)	O8—C24—C25	124.0 (4)
O3—C7—C6	127.5 (4)	O8—C24—C23	119.6 (3)
N1—C7—C6	108.4 (3)	C25—C24—C23	116.4 (3)
N1—C8—C9	111.4 (3)	C26—C25—C30	117.4 (4)
N1—C8—H8A	109.4	C26—C25—C24	124.0 (4)
C9—C8—H8A	109.4	C30—C25—C24	118.6 (3)
N1—C8—H8B	109.4	C27—C26—C25	120.6 (4)
C9—C8—H8B	109.4	C27—C26—Br2	115.6 (3)
H8A—C8—H8B	108.0	C25—C26—Br2	123.8 (3)
O4—C9—C10	119.6 (4)	C28—C27—C26	120.3 (4)
O4—C9—C8	119.7 (3)	C28—C27—H27	119.9
C10—C9—C8	120.6 (3)	C26—C27—H27	119.9
C11—C10—C15	117.1 (4)	C29—C28—C27	120.0 (4)
C11—C10—C9	128.4 (4)	C29—C28—H28	120.0
C15—C10—C9	114.5 (4)	C27—C28—H28	120.0
C10—C11—C12	121.6 (4)	C28—C29—C30	119.9 (4)
C10—C11—Br1	123.4 (3)	C28—C29—H29	120.0
C12—C11—Br1	114.8 (3)	C30—C29—H29	120.0
C13—C12—C11	119.3 (4)	C29—C30—C25	121.8 (4)
C13—C12—H12	120.4	C29—C30—H30	119.1
C11—C12—H12	120.4	C25—C30—H30	119.1
			
O1—S1—N1—C7	−111.1 (3)	Br1—C11—C12—C13	175.6 (3)
O2—S1—N1—C7	119.3 (3)	C11—C12—C13—C14	1.1 (7)
C1—S1—N1—C7	4.1 (3)	C12—C13—C14—C15	−1.1 (7)
O1—S1—N1—C8	65.3 (3)	C13—C14—C15—C10	0.4 (7)
O2—S1—N1—C8	−64.3 (3)	C11—C10—C15—C14	0.2 (6)
C1—S1—N1—C8	−179.6 (3)	C9—C10—C15—C14	−179.4 (4)
O5—S2—N2—C22	−116.7 (3)	O5—S2—C16—C21	113.1 (3)
O6—S2—N2—C22	113.0 (3)	O6—S2—C16—C21	−112.2 (3)
C16—S2—N2—C22	−1.8 (3)	N2—S2—C16—C21	0.3 (3)
O5—S2—N2—C23	70.9 (3)	O5—S2—C16—C17	−67.8 (4)
O6—S2—N2—C23	−59.4 (3)	O6—S2—C16—C17	66.9 (4)
C16—S2—N2—C23	−174.2 (3)	N2—S2—C16—C17	179.4 (3)
O1—S1—C1—C2	−69.0 (4)	C21—C16—C17—C18	−0.4 (6)
O2—S1—C1—C2	65.8 (4)	S2—C16—C17—C18	−179.4 (3)
N1—S1—C1—C2	178.2 (4)	C16—C17—C18—C19	−0.8 (6)
O1—S1—C1—C6	109.9 (3)	C17—C18—C19—C20	1.5 (6)
O2—S1—C1—C6	−115.4 (3)	C18—C19—C20—C21	−0.9 (6)
N1—S1—C1—C6	−2.9 (3)	C17—C16—C21—C20	1.0 (6)
C6—C1—C2—C3	0.1 (6)	S2—C16—C21—C20	−179.9 (3)
S1—C1—C2—C3	178.8 (3)	C17—C16—C21—C22	−178.0 (3)
C1—C2—C3—C4	−0.5 (6)	S2—C16—C21—C22	1.1 (4)
C2—C3—C4—C5	0.3 (6)	C19—C20—C21—C16	−0.3 (5)
C3—C4—C5—C6	0.4 (6)	C19—C20—C21—C22	178.6 (4)
C4—C5—C6—C1	−0.8 (6)	C23—N2—C22—O7	−4.5 (6)
C4—C5—C6—C7	179.7 (4)	S2—N2—C22—O7	−176.9 (3)
C2—C1—C6—C5	0.6 (6)	C23—N2—C22—C21	175.0 (3)
S1—C1—C6—C5	−178.3 (3)	S2—N2—C22—C21	2.6 (4)
C2—C1—C6—C7	−179.9 (4)	C16—C21—C22—O7	177.2 (4)
S1—C1—C6—C7	1.3 (4)	C20—C21—C22—O7	−1.7 (6)
C8—N1—C7—O3	1.4 (6)	C16—C21—C22—N2	−2.3 (4)
S1—N1—C7—O3	177.7 (3)	C20—C21—C22—N2	178.8 (4)
C8—N1—C7—C6	179.8 (3)	C22—N2—C23—C24	83.4 (4)
S1—N1—C7—C6	−3.9 (4)	S2—N2—C23—C24	−104.7 (3)
C5—C6—C7—O3	−0.7 (7)	N2—C23—C24—O8	6.4 (5)
C1—C6—C7—O3	179.8 (4)	N2—C23—C24—C25	−172.9 (3)
C5—C6—C7—N1	−179.0 (4)	O8—C24—C25—C26	−7.6 (6)
C1—C6—C7—N1	1.5 (4)	C23—C24—C25—C26	171.7 (4)
C7—N1—C8—C9	90.7 (4)	O8—C24—C25—C30	171.8 (4)
S1—N1—C8—C9	−85.4 (4)	C23—C24—C25—C30	−8.9 (5)
N1—C8—C9—O4	−22.3 (5)	C30—C25—C26—C27	2.2 (6)
N1—C8—C9—C10	155.1 (3)	C24—C25—C26—C27	−178.4 (4)
O4—C9—C10—C11	−155.3 (4)	C30—C25—C26—Br2	−176.7 (3)
C8—C9—C10—C11	27.3 (6)	C24—C25—C26—Br2	2.7 (5)
O4—C9—C10—C15	24.3 (5)	C25—C26—C27—C28	−2.9 (6)
C8—C9—C10—C15	−153.1 (4)	Br2—C26—C27—C28	176.0 (3)
C15—C10—C11—C12	−0.2 (6)	C26—C27—C28—C29	1.4 (6)
C9—C10—C11—C12	179.4 (4)	C27—C28—C29—C30	0.7 (6)
C15—C10—C11—Br1	−176.0 (3)	C28—C29—C30—C25	−1.4 (6)
C9—C10—C11—Br1	3.6 (6)	C26—C25—C30—C29	−0.1 (6)
C10—C11—C12—C13	−0.5 (6)	C24—C25—C30—C29	−179.5 (4)

### Hydrogen-bond geometry (Å, º)

**Table:**

*D*—H···*A*	*D*—H	H···*A*	*D*···*A*	*D*—H···*A*
C3—H3···O7^i^	0.95	2.56	3.238 (5)	129
C14—H14···O1^ii^	0.95	2.40	3.305 (5)	159
C17—H17···O5^iii^	0.95	2.43	3.225 (5)	141
C27—H27···O7^iv^	0.95	2.29	3.164 (5)	153
C30—H30···O2^v^	0.95	2.51	3.251 (5)	135
C8—H8*A*···Br1	0.99	2.82	3.165 (4)	101
C23—H23*A*···O3	0.99	2.48	3.014 (5)	114

Symmetry codes: (i) −*x*+2, −*y*, −*z*; (ii) −*x*+2, −*y*+1, −*z*; (iii) −*x*+1, −*y*+1, −*z*+1; (iv) −*x*+2, −*y*, −*z*+1; (v) −*x*+1, −*y*, −*z*.
